# The prevalence and clinical features associated of hypothyroidism among Thai systemic sclerosis patients

**DOI:** 10.1038/s41598-021-94371-6

**Published:** 2021-07-21

**Authors:** Yathao Paolee, Chingching Foocharoen, Suranut Charoensri, Mayfong Mayxay, Ajanee Mahakkanukrauh, Siraphop Suwannaroj, Ratanavadee Nanagara

**Affiliations:** 1 Department of Medicine, Faculty of Medicine, Khon Kaen University, Khon Kaen, 40002 Thailand; 2 Lao-Oxford-Mahosot Hospital-Wellcome Trust-Research Unit, Mahosot Hospital, University of Health Sciences of Laos, Vientiane, Laos

**Keywords:** Endocrinology, Rheumatology

## Abstract

Thyroid disease, particularly hypothyroidism, has been reported in systemic sclerosis (SSc). Some clinical features of SSc can also present in hypothyroidism. Our aims were to determine the prevalence of, and describe clinical features associated with, hypothyroidism in SSc patients. We conducted a historical cohort study of adult SSc patients who underwent screening thyroid function tests at the Scleroderma Clinic, Khon Kaen University, Thailand, between 2009 and 2018. The patients who had any thyroid disorders before the onset of SSc and were diagnosed as an overlap syndrome were excluded. A total of 200 SSc were included according to sample size calculation, among whom the female to male ratio was 2:1. The majority of cases (137; 69.5%) were diffuse cutaneous SSc subset. The mean age was 55.8 ± 10.7 years and the median duration of disease 4.9 (IQR 1.6–9.9) years. Of the total, 9 had primary hypothyroidism (prevalence 4.5%; 95%CI 2.1–8.4) and 22 had subclinical hypothyroidism (prevalence 11%; 95%CI 7.0–16.2). Of the latter 22, 71% had dcSSc. Logistic regression analysis indicated that unexplained anemia was significantly associated with either subclinical hypothyroid or hypothyroidism (OR 2.74; 95% CI 1.17–6.47), whereas Raynaud’s phenomenon had a negative association (OR 0.28; 95% CI 0.11–0.66). Neither severity of skin tightness nor internal organ involvement were associated with hypothyroidism among SSc patients. Clinical-subclinical hypothyroidism is uncommon among SSc patients, it is frequently associated with anemia, and less so Raynaud’s phenomenon. Clinical-subclinical hypothyroidism should thus be considered in cases of unexplained anemia in SSc patients.

## Introduction

Systemic sclerosis (SSc) is a connective tissue disease of unknown cause and complex pathogenesis. The disease includes 4 hallmarks: (a) auto-immunity, (b) inflammation, (c) functional and structural alterations in small blood vessels, and (d) widespread interstitial and vascular fibrosis affecting multiple organ systems. SSc seemingly confined to the skin, also involves visceral organs (kidneys, lungs, heart, and gastrointestinal tract) and trends to have significant morbidity and mortality for patients with SSc^1,2^.

Endocrine dysfunction can complicate the treatment process of patients with SSc. The most common endocrine problem associated with SSc is thyroid disease, and the frequency of other endocrine disorders is similar to that of the general population^2^. Several studies have documented that thyroid disease occurs in 10% to 14% of patients with SSc due to severe fibrosis of the thyroid gland^3–6^. Autoimmune regulator gene polymorphism has been linked to SSc in association with Hashimoto’s thyroiditis, and autoimmune thyroid disease seems to be associated with a higher index level of anti-Scl70^7,8^. A previous study revealed that SSc patients with diffuse cutaneous systemic sclerosis (dcSSc) had higher thyroid stimulating hormone (TSH) serum levels than that in patients with limited cutaneous systemic sclerosis (lcSSc). The TSH level was higher in SSc patients the more severe the skin disease, and TSH level was significantly correlated the higher the modified Rodnan’s skin score (mRSS) (p < 0.001 and r^2^ = 0.32) compared to those with normal TSH serum levels^9^.

Several studies have illustrated the high incidence and prevalence of new cases of hypothyroidism among SSc patients. Those studies demonstrated a close association between hypothyroidism in SSc and subcutaneous calcinosis, Raynaud’s phenomenon (RP), esophageal hypomotility, sclerodactyly, and multiple telangiectasia^2–5^. Shahin et al. found that an overall decrease in free thyroxin (FT4) levels among SSc patients with disease duration under 3 years correlated significantly with the DLCO level (r =  + 0.90, p < 0.01), while the FT4 level was significantly correlated to restricted motion of hand joints (r = 0.66, p < 0.01) in SSc patients with a disease duration over 3 years and having hypothyroidism^10^. Toki et al. showed the prevalence of anti-centromere antibody positivity and severe facial skin sclerosis among female SSc patients was significantly associated with the presence of autoimmune thyroiditis (AITD)^3^.

In clinical practice, the current recommendation for monitoring thyroid function in SSc patients is inconsistent. The aims of the current study were to determine the prevalence of hypothyroidism in Thai SSc and to describe clinical features of SSc associated with hypothyroidism.

## Materials and methods

This was a historical cohort study that included adult participants over 18 diagnosed with SSc at the Scleroderma Clinic, Khon Kaen University, Thailand, between 2009 and 2018. Evaluations (including thyroid function tests) were stored in the Scleroderma cohort database. We excluded participants diagnosed with any thyroid disorders before onset of SSc and the patients who were diagnosed SSc overlap with other connective tissue diseases.

A diagnosis of SSc was based on the criteria from the American Rheumatology Association (ARA) 1980 or the American College of Rheumatology and the European League Against Rheumatism (ACR/EULAR) 2013 Classification Criteria of SSc^11,12^. Duration of disease was based on the duration from onset of first non-Raynaud’s phenomenon symptom to the date of the thyroid function test. Table 1 shows the definition of clinical parameters determined from medical records and the criteria for diagnosis of thyroid status.

**Table 1:** Definition of clinical parameters determined from medical records and criteria for diagnosis of thyroid.

Medical term	Definition
Primary hypothyroidism	TSH > 4.2 mIU/L with FT4 < 0.93 ng/dL
Subclinical hypothyroidism	TSH > 4.2 mIU/L with FT4 within normal range^a^
Primary hyperthyroidism	TSH < 0.2 mIU/L with FT4 > 1.7 ng/dL and/or FT3 > 6.9 pg/mL
Subclinical hyperthyroidism	TSH < 0.2 mIU/L with FT4 and FT3 within the normal range^a^
Esophageal involvement	Any esophageal symptoms of SSc included esophageal dysphagia, heartburn, or reflux symptoms
Stomach involvement	Symptoms included dyspepsia, early satiety, and bloating
Pulmonary fibrosis	Interstitial fibrosis is detected by high resolution computed tomography (HRCT) of the lungs
Pulmonary arterial hypertension (PAH)	Defines as detected by right heart catheterization, mean pulmonary arterial pressure > 20 mmHg at rest, pulmonary capillary wedge pressure < 15 mmHg, and pulmonary venous resistance > 3 Wood units
Anemia	Hemoglobin < 12 g/dL in females and < 13 g/dL in males with excluding explained causes

TSH thyroid stimulating hormone; FT3 free triiodothyronine; FT4 free thyroxine.

^a^Normal range of FT4 0.93–1.7 ng/dL, FT3 2.3–6.9 pg/mL, TSH 0.2–4.2 mIU/L.

### Sample size

The sample size was calculated in light of the previous prevalence of hypothyroidism reported in the literature. The prevalence of hypothyroidism in SSc was 10–14%^3–6^. To determine the primary outcome of the study with 95% power at a significance level of 0.05, the sample size needed to be 183. We thus included 200 patients into the study.

### Statistical analysis

The data were described using frequencies and percentages. The prevalence of hypothyroidism in SSc was described with its 95% confidence interval (95%CI). Exploratory univariable analysis was done using univariable logistic regression. The odds ratio (OR) with 95%CI and p-value were used to determine the association between SSc clinical characteristics and clinical or subclinical hypothyroidism. Multivariable logistic regression analysis was then performed to identify clinical association with clinical or subclinical hypothyroidism. The removal of non-contributing associated factors was based primarily on clinical relevancy and statistical significance. Clinical variables with OR closest to 1.00 and insignificant P value of > 0.1 were sequentially eliminated from multivariable logistic regression model^13^. A P-value < 0.05 was considered statistically significant. All data analyses were performed using STATA version 16.0 (Stata Corp, College Station, TX).

The study was designed by the authors and approved by the Human Research Ethics Committee of Khon Kaen University per the Helsinki Declaration and the Good Clinical Practice Guidelines (HE61156). All eligible patients signed informed consent before enrollment in the cohort study. The sponsor had no role in the study.

### Ethical consideration

The Human Research Ethics Committee of Khon Kaen University reviewed and approved the study per the Helsinki Declaration and the Good Clinical Practice Guidelines (HE611156).

### Consent for publication

All of the authors consent to publishing and hereby grant the Publisher exclusive license of the full copyright.

## Results

A total of 200 medical records of SSc patients tested for thyroid function were reviewed. The female to male ratio was 2:1. The majority of cases (137; 69.5%) had dcSSc. The mean age was 55.8 ± 10.7 years and the median duration of the disease was 4.9 (IQR 1.6–9.9) years. Of the total, 9 cases had primary hypothyroidism for a prevalence of 4.5% (95%CI 2.1–8.4) and 22 had subclinical hypothyroidism for a prevalence of 11% (95%CI 7.0–16.2). Three cases (1.5%; 95%CI 0.3–4.3) were diagnosed with either hyperthyroidism (2 cases) or subclinical hyperthyroidism (1 case). The prevalence of primary hypothyroidism and subclinical hypothyroidism among Thai SSc patients was 15.5% (95%CI 10.8–21.3). Of the patients who had either hypothyroidism or subclinical hypothyroidism, 22 cases (71%) had the dcSSc subset, and 13 (41.9%) had a disease duration under 4 years. Two cases (6.5%) had a body mass index (BMI) under 18.5 kg/m^2^.

Due to the low number of cases of both clinical and subclinical hyperthyroidism, we excluded the condition from the analysis and due to the low number of cases of primary and subclinical hypothyroidism, we combined them both into the clinical-subclinical hypothyroidism group. The clinical association of clinical-subclinical hypothyroidism in SSc according to the univariate analysis revealed that RP, weight loss, and anemia were associated with clinical-subclinical hypothyroidism with a respective OR of 0.25 (95%CI 0.09–0.61), 0.28 (95%CI 0.05–0.99), and 2.48 (95%CI 1.03–6.27). Onset duration, the severity of skin tightness, internal organ involvement and serology were not associated with clinical-subclinical hypothyroidism in SSc patients (Table 2).

**Table 2:** Clinical parameters associated with clinical-subclinical hypothyroidism.

Data	Normal thyroid hormone N = 166	Clinical–subclinical hypothyroidism N = 31	Odds ratio (95%CI)	P-value
Age at onset > 60 years	26 (15.7)	6 (19.4)	1.30 (0.40–3.60)	0.60
Duration of disease ≤ 4 years, N (%)	76 (45.8)	13 (41.9)	0.86 (0.36–2.00)	0.84
Female, N (%)	107 (64.5)	25 (80.6)	2.30 (0.85–7.21)	0.09
dcSSc subset, N (%)	115 (69.3)	22 (70.9)	1.08 (0.45–2.86)	1.00
BMI < 18.5 kg/m^2^, N (%)	14 (29.2)	2 (28.5)	0.97 (0.08–6.84)	1.00
mRSS > 20 points, N (%)	17 (10.2)	4 (12.9)	1.3 (0.30–4.41)	0.75
**Clinical symptoms on the study date**				
RP, N (%)	103 (62.2)	9 (29.0)	0.25 (0.09–0.61)	0.001*
Digital ulcer, N (%)	41 (24.7)	5 (16.1)	0.61 (0.17–1.79)	0.36
Digital gangrene, N (%)	1 (0.6)	0	NA	
Telangiectasia, N (%)	36 (21.7)	9 (29.0)	1.5 (0.56–3.78)	0.36
Calcinosis cutis, N (%)	5 (3.1)	2 (6.4)	2.19 (0.20–14.10)	0.30
Salt and pepper skin, N (%)	66 (40.2)	12 (38.7)	0.93 (0.38–2.19)	1.00
Edematous skin, N (%)	27 (16.5)	7 (22.5)	1.48 (0.49–1.98)	0.44
Tendon friction rub, N (%)	22 (13.4)	2 (6.4)	0.44 (0.04–1.98)	0.38
Hand deformity, N (%)	50 (30.5)	10 (32.2)	1.08 (0.42–2.62)	0.83
Arthritis, N (%)	14 (8.5)	2 (6.4)	0.73 (0.07–3.50)	1.00
Muscle weakness, N (%)	14 (8.5)	0	NA	
Esophageal involvement, N (%)	58 (35.4)	13 (41.9)	1.32 (0.55–3.08)	0.54
Stomach involvement, N (%)	29 (17.7)	6 (19.3)	1.11 (0.34–3.13)	0.80
Constipation, N (%)	30 (18.3)	5 (16.1)	0.85 (0.23–2.53)	1.00
Weight loss, N (%)	45 (27.4)	3 (9.6)	0.28 (0.05–0.99)	0.04*
Pulmonary fibrosis, N (%)	66 (39.7)	8 (25.8)	0.52 (0.19–1.31)	0.16
PAH, N (%)	14 (8.4)	0	NA	
**Laboratory parameters**				
ATA, N = 39 (%)	31 of 34 (91.1)	3 of 5 (60.0)	0.14 (0.01–2.56)	0.11
ACA, N = 24 (%)	1 of 24 (4.1)	1 of 2 (50.0)	23 (0.14–1877.70)	0.15
Anemia, N (%)	76 (45.7)	21 (67.7)	2.48 (1.03–6.27)	0.03*
FVC < 70%, N (%)	48 (55.1)	5 (71.4)	2.03 (0.30–22.20)	0.46
Pericardial effusion (present), N (%)	14 (14.4)	2 (14.2)	0.98 (0.09–5.22)	1.00
LVEF (%) < 50%, N (%)	4 (4.0)	0	NA	

CI: confidence interval; dcSSc: diffuse cutaneous systemic sclerosis; BMI: body mass index; RP: Raynaud’s phenomenon; PAH: pulmonary arterial hypertension; ATA: anti-topoisomerase I antibody; ACA: anti-centromere antibody; FVC forced vital capacity; LVEF left ventricular ejection fraction; NA: data not available due to a statistical limitation.

*Statistical significance;

After logistic regression analysis, anemia was associated with clinical-subclinical hypothyroidism with an adjusted OR of 2.74 (95%CI 1.17–6.47), while RP had a negative association with clinical-subclinical hypothyroidism with an adjusted OR of 0.28 (95%CI 0.11–0.66) (Table 3.).

**Table 3:** Logistic regression analysis of clinical parameter of SSc associated with clinical-subclinical hypothyroidism.

Factor	Crude OR (95%CI)	Adjusted OR (95%CI)	p-value
Female	2.30 (0.85–7.21)	2.11 (0.78–5.71)	0.14
Raynaud’s phenomenon	0.25 (0.09–0.61)	0.28 (0.11–0.66)*	0.004*
Weight loss	0.28 (0.05–0.99)	0.31 (0.08–1.10)	0.07
Anemia	2.48 (1.03–6.27)	2.74 (1.17–6.47)*	0.02*

CI: confidence interval; OR: odds ratio.

*Statistically significant.

## Discussion

Thyroid disease is uncommon in SSc. According to our observations, 34 cases (17%) of our patients had an abnormal thyroid function test, of whom 1% had hyperthyroidism, 0**.**5% had subclinical hyperthyroidism, 4**.**5% had hypothyroidism, and 11% had subclinical hypothyroidism**.** The prevalence of hypothyroidism among our SSc patients was less than that in previous studies, where the prevalence of primary hypothyroidism was between 10 and 14% in SSc (13.8% in Japan^3^, 10–14% in the United States^4,5^, and 9.4% in New Zeeland^6^). The difference in prevalence might be explained by differences in the mean age of participants among the studies. The mean age of our participants was 55.8 ± 10.7 years vs. 64.9 ± 0.7 years in a previous study^3^. Generally, hypothyroidism trends higher in the older persons^14^. Autoimmune disease (Hashimoto’s thyroiditis)—also more common with age—exacerbates the potential for hypothyroidism^15^. Another possible reason for the low prevalence of hypothyroidism in our study is the short disease duration of our patients compared to that of previous studies. The median duration of SSc in our study population was 4.9, while it was 8 years from the first non-Raynaud’s phenomenon symptom in the previous study^9^. Longer disease duration might be associated with higher prevalence of hypothyroidism because of the increasing rate of thyroid gland fibrosis. Other reasons for the low prevalence of hypothyroidism in our study might be ethnic differences or genetic factors^16^.

The presence of anemia was strongly associated with the presence of clinical-subclinical hypothyroidism among our Thai SSc patients. The patients who had anemia trended to have clinical-subclinical hypothyroidism 2.7 times more than those who had no anemia. Our finding agrees with previous studies^17,18^. Kosenli et al. reported the prevalence of anemia in the hypothyroidism group was 43%, subclinical hypothyroidism group 39%, vs. 26% in the control group. The prevalence of anemia in both the hypothyroidism group and subclinical hypothyroidism group was significantly higher than in the control group (p = 0.003 and p = 0.021, respectively)^18^. The association between hypothyroidism and anemia has been postulated as being due to absent or insufficient thyroid hormones for erythropoiesis stimulation^19^. Anemia due to erythropoeitin insufficiency or deficiency is normochromic normocytic anemia. The anemia in hypothyroidism should then be normochromic normocytic^20^; however, normocytic, hypochromic-microcytic, or macrocytic anemia have all been reported in hypothyroidism^21^. Clinical-subclinical hypothyroidism should thus be considered in cases of unexplained anemia in SSc patients and a thyroid function test should be done to evaluate such patients.

The presence of RP was inversely associated with clinical-subclinical hypothyroidism in our SSc patients. Not only RP but also other signs of vasculopathy. In particular, digital ulcer, digital gangrene, and PAH were less frequently observed in SSc with clinical-subclinical hypothyroidism than among those with a normal thyroid function test. Since none of the patients with clinical-subclinical hypothyroidism had digital gangrene or PAH, there was a statistical limitation to determine a negative association between vasculopathy and clinical-subclinical hypothyroidism. Nevertheless, the negative association between vasculopathy and hypothyroidism in our study contrasts with other studies in which the presence of RP was found in those who had hypothyroidism. Most reports are case reports and do not include SSc patients. This is because RP has been reported in patients with hypothyroidism and symptoms disappeared after thyroid replacement therapy^22,23^. RP with Graves’ disease has been reported as a presentation of lcSSc for many years before lcSSc was diagnosed^24^. Withrington et al. also reported a patient with a diagnosis of mixed connective tissue disease and that RP developed hypothyroidism 15 months later^25^. There is also a case report of a patient who presented with clinical myocardial infarction but a normal coronary angiogram showed coexisting RP and thyroid deficiency^26^. Until recently, the association between RP and thyroid disease was uncertain. Due to the low prevalence of hypothyroidism among our SSc patients, the sample size is insufficient to confirm the actual association between vasculopathy and hypothyroidism. We postulate that the result might be due to chance since the prevalence of RP was high in SSc compared to other SSc clinical features, and that the prevalence waxes and wanes according to the temperature. RP is usually detected after contact with the cold, so the prevalence of RP might have been over or underestimated depending on the time of record. If the result did not occur by chance, it might be assumed that vasculopathy is not a principal of pathogenesis nor does it play a major role of thyroid dysfunction in SSc. Nailfold capillaroscope is a tool for evaluating microcirculation and microvascular abnormality in SSc. It can stratify RP and might be helpful for evaluating the severity of RP^27^. Because the capillaroscope was not our routine investigation and we have facility limitation (experience of the operator and budget), so we cannot provide the information of nailfold pattern evaluating by capillaroscope among SSc with and without hypothyroidism. To clarify the association between RP and hypothyroidism in SSc patients, further study in a larger population as well as a vascular study (i.e., nailfold capillaroscope, Doppler ultrasound of thyroid gland in SSc patients with hypothyroidism) is suggested.

The clinical features associated with clinical-subclinical hypothyroidism among SSc patients vary according to population-based studies^3,5,6,9^. Some reported that severe skin tightness^9^ or anti-topoisomerase antibody (ATA)^6^—which are associated with severe disease—are commonly detected in SSc patients with hypothyroidism, while other researchers reported a high prevalence of a mild form of the disease (CREST syndrome)^5^ or anti-centromere antibody^3^ among patients with hypothyroidism. A comparison of the prevalence and associated clinical features of clinical-subclinical hypothyroidism in SSc patients found in the literature and our study are presented in Table 4. Per the literature review, a clinical association with hypothyroidism in SSc remains inconclusive.

**Table 4:** Comparison of prevalence and clinical features associated with clinical-subclinical hypothyroidism among SSc patients.

Authors	N	Ethnic	Clinical-subclinical hypothyroidism			Clinical association
			Overall	dcSSc	lcSSc	
Our study	200	Thai	15.5%	71%	29.0%	High prevalence of anemia in clinical-subclinical hypothyroidism Low prevalence of RP in clinical-subclinical hypothyroidism
Toki et al.^3^	210	Japanese	13.8%	20%	80%	High prevalence of ACA positivity, Sjogren’s syndrome, and severe facial skin sclerosis in AITD
Gordon et al.^5^	56	Pennsylvania	14% hypothyroidism			High prevalence of CREST syndrome in hypothyroidism
Solanki et al.^6^	85	New Zealand	9.4% subclinical hypothyroidism			Positive for both anti-Tg and anti-TPO antibodies, with primary hypothyroidism
Bagnato et al.^9^	105	Italian				Severe skin thickness by mRSS and high TSH level
Shahin et al.^10^	23	Egyptian				Correlation between hand joint restriction of motion and hypothyroidism in SSc patients with disease duration over 3 years
Marasini et al.^34^	40	Italian				SSc patients with hypothyroidism had higher pulmonary arterial pressure over against SSc patients with hyperthyroidism (46 vs. 37 mmHg)
Danielides et al.^35^	138	Grecian				High prevalence of anti-TPO in lcSSc

dcSSc: diffuse cutaneous systemic sclerosis; lcSSc: limited cutaneous systemic sclerosis; RP: Raynaud’s phenomenon; ACA: Anti-centromere antibody; CREST syndrome: calcinosis, Raynaud's phenomenon, esophageal dysmotility, sclerodactyly, and telangiectasia. anti-Tg: antithyroglobulin; anti-TPO: anti-thyroid peroxidase antibodies.

The possible cause of hypothyroidism in SSc is claimed to be due to autoimmune thyroiditis (AITD), especially Hashimoto’s thyroiditis. The exact mechanism of autoimmune thyroiditis in SSc is not known, but it is thought that both genetic and environmental factors are involved and play roles vis-à-vis the presence of ATA in SSc patients^28^. Bliddal et al.^29^ reported that the mechanisms of natural polyautoimmunity would play a role in the presence of autoantibodies and the loss of self-tolerance in the immune system. In addition, AITD is often present in patients with polyautoimmunity, so patients with AITD might have an increased risk of developing autoimmune diseases. A Colombian study included 1083 patients for investigation the cluster patterns of polyautoimmunity. The authors found that SSc was the most common disease that coexists with AITD (23%), followed by rheumatoid arthritis (21%), systemic lupus erythematosus (18%), and multiple sclerosis (9%)^30^. Another study of 179 Italian SSc patients with 179 age- and gender-matched control subjects revealed a high incidence of new cases of hypothyroidism, thyroid dysfunction, and anti-thyroperoxidase antibody positivity in SSc patients with a respective incidence of 15.5, 21, and 11 per 1000 patient-years. The logistic regression analysis from the same study showed an association between borderline high TSH and anti-thyroperoxidase antibody positivity in SSc patients^31^. Although AITD is likely a cause of hypothyroidism in SSc, we were unable to test for thyroid antibody, so we cannot clarify the etiology of hypothyroidism in our SSc patients and cannot determine the respective prevalence of AITD.

Another possible mechanism of hypothyroid in SSc would be related to fibrosis. A previous study reported on the association between high mRSS and high TSH level^9^. In it, Bagnato et al.^9^ reported that in an age- and sex-matched control autopsy series, 56 of the fatal SSc patients had histologic evidence of severe fibrosis of the thyroid glands (i.e., 14% vs. 2% of pathologic vs. serologic cases).All of thyroid tissue of SSc patients with hypothyroidism had fibrosis with some lymphocytic infiltration, and in 6 of 7 patients circulating anti-thyroglobulin antibody was also detected^5^. The authors concluded that the thyroid autoimmune process led to gland fibrosis and hypothyroidism in severe SSc patients^5^. Our study did not find any association between the severity of fibrosis and clinical-subclinical hypothyroidism despite the fact that the majority of our SSc patients had the severe form SSc (dcSSc). Thus, the fibrosis mechanism for hypothyroidism in our SSc remains in question.

Since our study analysed the clinical association with a screening thyroid function test, none of the patients had any symptoms of either hyperthyroidism or hypothyroidism. Further study on the clinical outcomes of subclinical hypothyroidism—particularly the progression to primary hypothyroidism and the morbidity and mortality in SSc patients who had primary hypothyroidism—is recommended to determine the clinicals to monitor and the treatment guidance.

Our study had limitations due to the nature of retrospective data collection. We were thus unable to perform some investigations involving or affected by thyroid disease including pulmonary function tests and echocardiography on the same date of the thyroid function test evaluation. The tests were, however, mostly performed within 3–6 months before/after the thyroid function test assessment. No thyroid antibody was tested, so we cannot provide data on autoimmune thyroiditis in our patients. There were some missing data of ATA and anti-centromere antibody (ACA). Because neither anti-topoisomerase I nor ACA can help differentiating the SSc subset in Thai SSc patients^32^, so the autoantibodies are not always tested as a routine investigation among Thai SSc. Specific autoantibodies of SSc other than ATA and ACA (anti-RNA polymerase III, anti-Th/To) were also tested in minority of the patients. Due to the low prevalence of other related autoantibodies among Thai SSc and no clinical relevance^33^, we did not analyse the thyroid hormone status with those antibodies. Finally, the findings of this study were analysed from a single center, the results might be not applicable to the entire Thai population. Owing to the large study population and sample size calculation, there is a high level of confidence of the estimated prevalence of thyroid disease among Thai SSc patients. The preliminary data may provide a baseline for assisting in the evaluation of SSc patients and could be used for improving the care of SSc patients in daily practice.

## Conclusion

Clinical-subclinical hypothyroidism is rare in SSc patients. It is associated with a high frequency of anemia and a low frequency of Raynaud’s phenomenon. Clinical-subclinical hypothyroidism should thus be considered in cases of unexplained anemia in SSc patients.

## Data Availability

Data and materials are available upon request.
